# A combinational feature selection and ensemble neural network method for classification of gene expression data

**DOI:** 10.1186/1471-2105-5-136

**Published:** 2004-09-27

**Authors:** Bing Liu, Qinghua Cui, Tianzi Jiang, Songde Ma

**Affiliations:** 1 National Laboratory of Pattern Recognition, Institute of Automation, Chinese Academy of Sciences, Beijing 100080, P. R. China

## Abstract

**Background:**

Microarray experiments are becoming a powerful tool for clinical diagnosis, as they have the potential to discover gene expression patterns that are characteristic for a particular disease. To date, this problem has received most attention in the context of cancer research, especially in tumor classification. Various feature selection methods and classifier design strategies also have been generally used and compared. However, most published articles on tumor classification have applied a certain technique to a certain dataset, and recently several researchers compared these techniques based on several public datasets. But, it has been verified that differently selected features reflect different aspects of the dataset and some selected features can obtain better solutions on some certain problems. At the same time, faced with a large amount of microarray data with little knowledge, it is difficult to find the intrinsic characteristics using traditional methods. In this paper, we attempt to introduce a combinational feature selection method in conjunction with ensemble neural networks to generally improve the accuracy and robustness of sample classification.

**Results:**

We validate our new method on several recent publicly available datasets both with predictive accuracy of testing samples and through cross validation. Compared with the best performance of other current methods, remarkably improved results can be obtained using our new strategy on a wide range of different datasets.

**Conclusions:**

Thus, we conclude that our methods can obtain more information in microarray data to get more accurate classification and also can help to extract the latent marker genes of the diseases for better diagnosis and treatment.

## Background

With the successful completion of the Human Genome Project (HGP), we are entering the post genomic era. Facing mass amounts of data, traditional biological experiments and data analysis techniques encounter great challenges. In this situation, cDNA microarrays and high-density oligonucleotide chips are novel biotechnologies as global (genome-wide or system-wide) experimental approaches that are effectively used in systematical analysis of large-scale genome data. In recent years, with its ability to measure simultaneously the activities and interactions of thousands of genes, microarray promises new insights into the mechanisms of living systems and is attracting more and more interest for solving scientific problems and in industrial applications. Meanwhile, further biological and medical research also promoted the development and application of microarray.

Typical issues addressed by microarray experiments include two main aspects: finding co-regulated genes for classification based on different cell-type [1], stage-specific [2,3], disease-related [4-6], or treatment-related [6-8] patterns of gene expression and understanding gene regulatory networks by analyzing functional roles of genes in cellular processes [9,10]. Here we focus on the former, especially on tumor classification using gene expression data, which is a hot topic in recent years and has received general attention by many biological and medical researchers [11-19]. A reliable and precise classification of tumors based on gene expression data may lead to a more complete understanding of molecular variations among tumors, and hence, to better diagnosis and treatment strategies.

Microarray experiments usually generate large datasets with expression values for thousands of genes (2000~20 000) but not more than a few dozens samples (20~80). Thus, very accurate classification of tissue samples in such high-dimensional problems is difficult, but often crucial, for successful diagnosis and treatment. Several comprehensively comparative and improved methods have been proposed recently [20-22]. In this paper, we introduce a combinational feature selection method using ensemble neural networks to remarkably improve the accuracy and robustness of sample classification. In recent years, several researchers have used ensemble neural networks for tumor classification based on gene expression data [12,23]. Khan et al. [12] used neural networks to classify 4 subcategories of small round blue-cell tumors. By using 3750 networks generated by three fold cross-validation 1250 times and using the list of 96 most influential genes as the inputs, they reported very excellent results based on their dataset. Also O'Neill and Song [23] used neural networks to analyze lymphoma microarray data and can predict the long-term survival of individual patients with 100% accuracy based on the datasets published by Alizadeh et al [18]. Both of them are very good work in microarray data analysis using neural networks. In this paper our motivation lies in that by combining various feature selection mechanisms we can avail of more information of samples for classification and by using ensemble neural networks we can more effectively combine these features and improve the stability and robustness of answers. So the most important distinctions between our work and these above two citations are that by using combinational feature selection we can penetrate various different profiles of the samples and can avail of more information for classification, and also these neural networks can work in a parallel way unlike those two papers. In the same time, unlike their work based on some certain dataset, we can get improved, at least comparable results on a wide range of different datasets. In the following section, we provide detailed illustration and comparison of our new method.

## Results

### The general framework and implementation of our method

**Figure 1:**
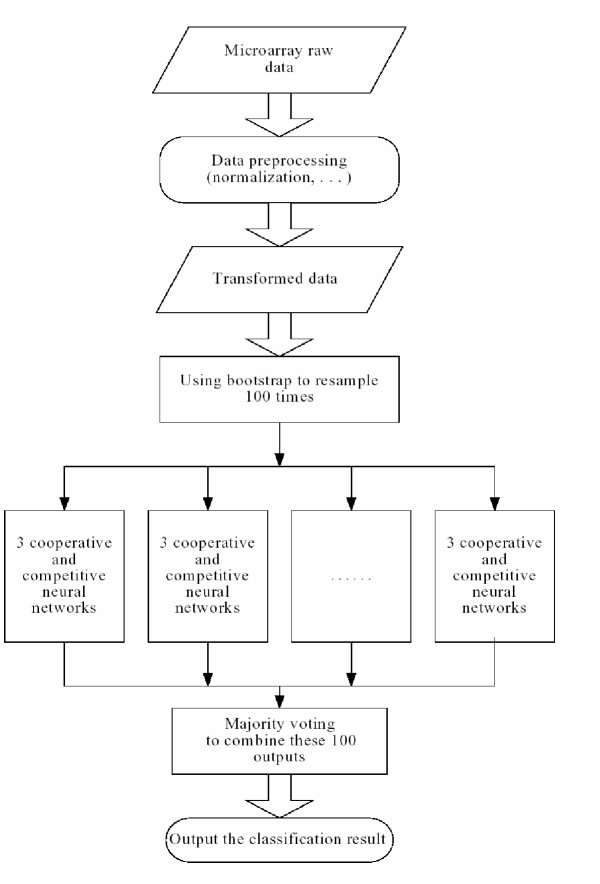
The whole flow chart

The flowchart of our method can be seen in Figure 1. When we obtain the microarray raw data based on a certain classification problem, first we need to preprocess them in order to be beneficial for further analysis. Broadly defined, pre-processing includes the planning and design of experiments, the acquisition and preprocessing of images, data transformation, data inspection, and data filtering. In this paper we avail of the publicly available datasets in , so we simplify this step and only use all datasets exactly as we found them in their transformed data.

**Figure 2:**
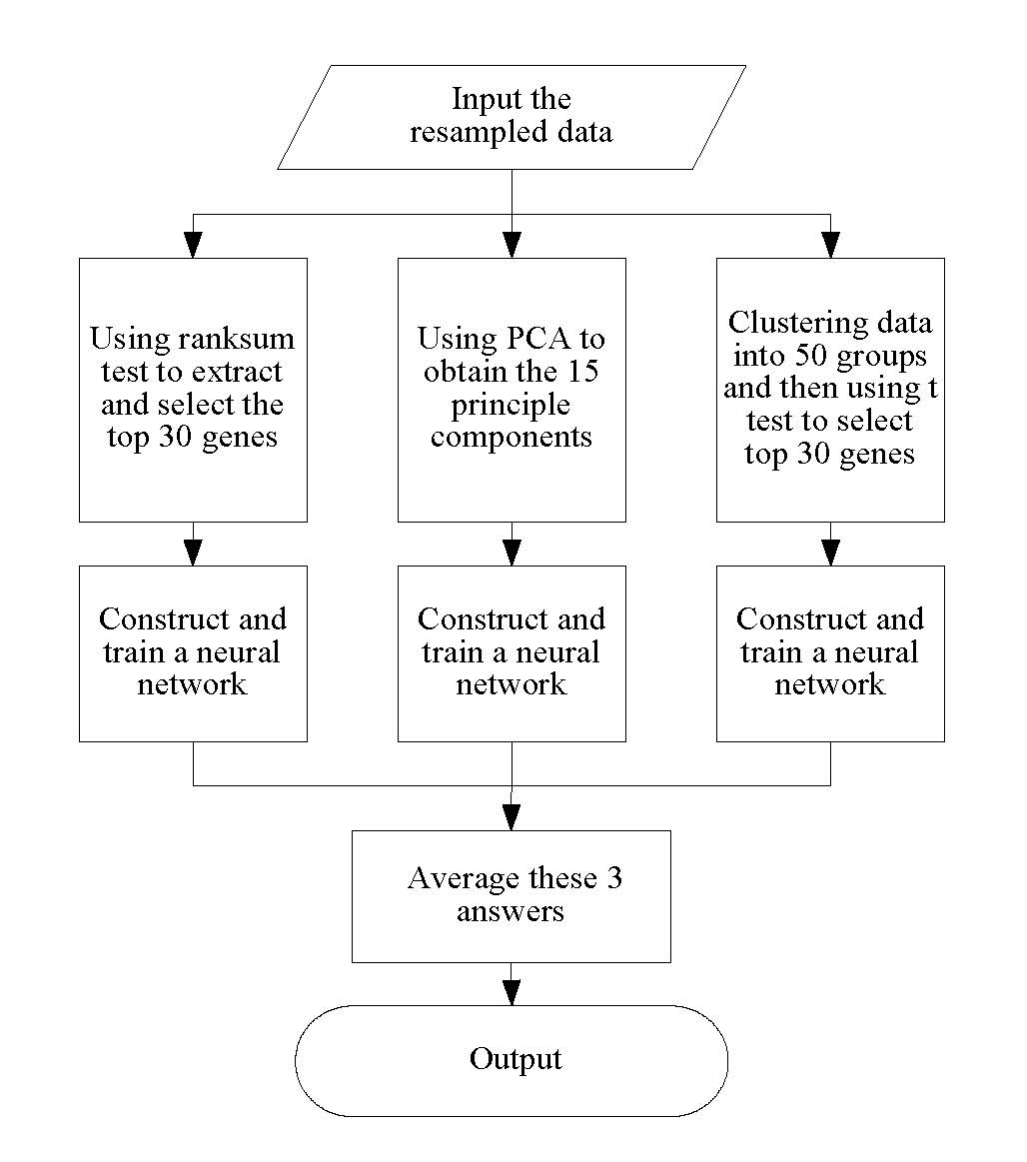
Three cooperative and competitive neural networks

Due to the characteristic of small sample numbers in microarray data, in order to improve the accuracy, robustness and generalization of issue classification, we apply bootstrap mechanism to resample 100 iterations. During each iteration, we input the resample training data into three cooperative and competitive neural networks, and then by averaging their decisions, the neural network set can output their discrimination. From Figure 2, we can clearly understand the architecture of these three neural networks. After obtaining the transformed resampling data, we extract and select features respectively based on ranksum test, PCA, clustering and t test. Ranksum test (also named Wilcoxon/Mann-Whitney test) is a nonparametric test, which does not take values into account and only calculates their scores purely based on rank information. We chose the top-ranked 30 genes identified as differentially expressed between the two types of tissues according to the ranksum test with the highest confidence (here using training data) as the first network input. At the same time, we used PCA to extract the principle components of all genes and used the top 15 principle components as the features to input another neural network. Also, we used Jaeger's "Masked out Clustering" ideas to group all the genes into 50 clusters and then used a t test to obtain the top 30 significant genes. Here we assume that each cluster can belong to the same pathway, genes which are co-expressed or are coming from the same chromosome. In this way, we can prefilter the gene set and drop genes that are very similar or highly correlated; that is, we can select the more significant genes for our discrimination as the third network input. More information about feature selection can be found in the methods section later. Based on these above three kinds of features we selected as the input, we construct and train three neural networks. Here we adopt simple one-hidden-layer feed-forward networks, which have 10 hidden units and one output unit for binary classification problem. As for multi-class problems, we can accordingly change the number of output units. Because each of these three networks adopts different feature selection mechanism as inputs, these inputs respectively reflect different aspects of samples, that is, different feature space in discriminative problems. We believe that this strategy of feature selection for issue classification reflects more profiles of different classes and will be able to obtain more accurate solution. Actually each of three networks is just like an expert holding a different judgment mechanism. Through averaging the confidences of three experts' answers, we can get the answer of this expert system. In this way, we not only can get the confidence of each expert, also we can judge the weight of each type of features in the answer. Finally, through competitive neural networks the robustness of this problem will be improved greatly.

After completing the 100 iterations, we can get 100 individual answers about the problem. In this situation, how to combine these answers into one more precise result is still a problem. Here, we simply use majority voting to combine the result and then give the ultimate solution about this classification problem. As noted above, here we adopt the soft-voting mechanism, that is, we can combine the confidence of each net. All the implementations of our framework were written in Matlab, using the hardware platform of a PC running 2.4 GHz.

### Datasets illustration

In this section, simple illustrations of the datasets we used in this paper for exploring the performance of our classification are given. The datasets in our paper have been downloaded from the following website: . We adopted their transformed data format for further research. All datasets we used can be reduced to three categories: binary class with testing samples, binary class without testing samples and multiple class problem. Here we classify samples into binary class with testing samples and without testing samples just according to the reference authors for each dataset. One important reason is that in this way we can easily compare our result with others based on the same training and testing sets. These datasets are shown in Table 1.

We use the three datasets below as the example of the first category, for which performance of our classification can be tested using the error ratio of testing samples.

#### ALL-AML leukemia

The training dataset consists of 38 bone marrow samples (27 ALL and 11 AML), with 7129 probes from 6817 human genes. Also, 34 samples testing data is provided, with 20 ALL and 14 AML.

More information and raw data can be found in Golub et al. [11].

#### Lung cancer

The dataset can be reduced to the problem of classification between malignant pleural mesothelioma (MPM) and adenocarcinoma (ADCA) of the lung. The training set contains 32 tissue samples, which consists of 16 MPM and 16 ADCA and the testing samples are constitutive of 15 MPM and 134 ADCA. Each sample is described by 12533 genes. More information about this dataset can be found in Gordon et al. [17].

#### Prostate cancer

For the prostate cancer dataset, detailed explanation and raw data is available in Singh et al. [5]. This dataset consists of 102 training vs. 34 testing (Tumor versus Normal classification) samples. The training set contains 52 prostate tumor samples and 50 normal samples with around 12600 genes and the independent test sets consist of 25 tumor and 9 normal samples.

Another three recently popular datasets have been used as the representative of the second category. Using these kinds of datasets, we apply cross-validation to validate our classification performance.

#### Types of diffuse large B-cell lymphoma

This dataset is used for discriminating distinct types of diffuse large B-cell lymphoma (DLBCL) using gene expression data. There are 47 samples, 24 of them are from "germinal canter B-like" group while the rest 23 are form "activated B-like" group and each sample can be described by 4026 genes. More detailed explanation can be found in Alizadeh et al. [18].

#### Ovarian cancer

The goal of this significant experiment is to identify proteomic patterns in serum that distinguish ovarian cancer from non-cancer. The proteomic spectra were generated by mass spectroscopy and the dataset provided here is 6-19-02, which includes 91 controls (Normal) and 162 ovarian cancers with 15154 molecular mass / charge (M/Z) identities. Here we use the transformed normalization data in . More information can be found in Petricoin et al. [6].

#### Colon tumor

The dataset Contains 62 samples collected from colon-cancer patients. Among them, 40 tumor biopsies are from tumors (labelled as "negative") and 22 normal (labelled as "positive") biopsies are from healthy parts of the colons of the same patients. Two thousand out of around 6500 genes were selected based on the confidence in the measured expression levels. Raw data and more information can be found in Alon et al. [14].

Finally, we can generalize our method from binary class to multi-class problems. In this paper, we evaluate the performance using the dataset below.

#### MLL_leukemia

This dataset contains training data consisting of 57 leukemia samples (20 ALL, 17 MLL and 20 AML) and testing data consisting of 4 ALL, 3 MLL and 8 AML samples. We adopted the transformed data from . More information can be seen in Armstrong et al. [15].

### Our results

First we primarily focus on the binary class problem. Because most of problems can be reduced to binary class problems, such as diseased vs. normal, survival vs. lethal, two opposite subtypes of some diseases and so on. Finally we generalize our classifier to multi-class application. In this paper, we evaluate the performance of different classification methods using predictive accuracy, which can be defined as:


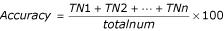


Here, *TN*1,*TN*2,…,*TNn *respectively denote the correct classification numbers of the samples belonging to a corresponding class; *totalnum *represents total sample numbers.

#### The results of binary classification with testing samples

For the first category of the datasets, we evaluate the performance of our classifiers using predictive accuracy of testing samples compared with the best performance of the current available methods. In this paper we use bagging to resample just as Tan and Gilbert [24], and we also compared our results to those using their bagged decision trees. In Table 2, we described the recognition rate of our methods compared with the best classifiers of our knowledge for each certain dataset and bagged decision trees proposed by Tan and Gilbert [24]. From this table, it is clear that our results are remarkably better than others based on these several datasets.

For the most popularly used AML-ALL leukemia dataset, to our knowledge, the best classifiers of this dataset can be found in [25-27], which can predict the results with 97.1% accuracy. However, we designed the classifiers using our methods based on 38 training samples, 0 error number of 34 testing samples can be obtained from our classifier.

In the same way, we trained our ensemble of neural networks using 32 training sets of lung cancer and then predicted the 149 separate testing sets still with 0 error number. And three (1:2) testing error numbers can be reached using methods by Li et al. [28], which is the best performance corresponding to this dataset of our knowledge.

For the third prostate cancer dataset, after training the classifier using 102 training sets, only one wrong classification can happen using our ensemble neural networks to predict the 34 separate testing samples. We did not find a more accurate classification result except for the bagged decision trees in [24] based on this dataset, so here we think that is the best result. In this sense, a great improvement in predictive accuracy can be obtained by using our method.

In order to further validate the effectiveness of predictive accuracy, we also performed the leave-one-out cross-validation (LOOCV) respectively only on the above three training samples. We also obtained the 100% accuracy both on the AML-ALL leukemia dataset and the lung cancer dataset, which are the same results as using individual testing samples. At the same time, 96.08% accuracy can be got based on the prostate cancer dataset, which is a little lower than using individual testing samples. For the purpose of comparison, we also list these results in Table 2. Thus, we conclude that our performance evaluation is credible.

**Figure 3:**
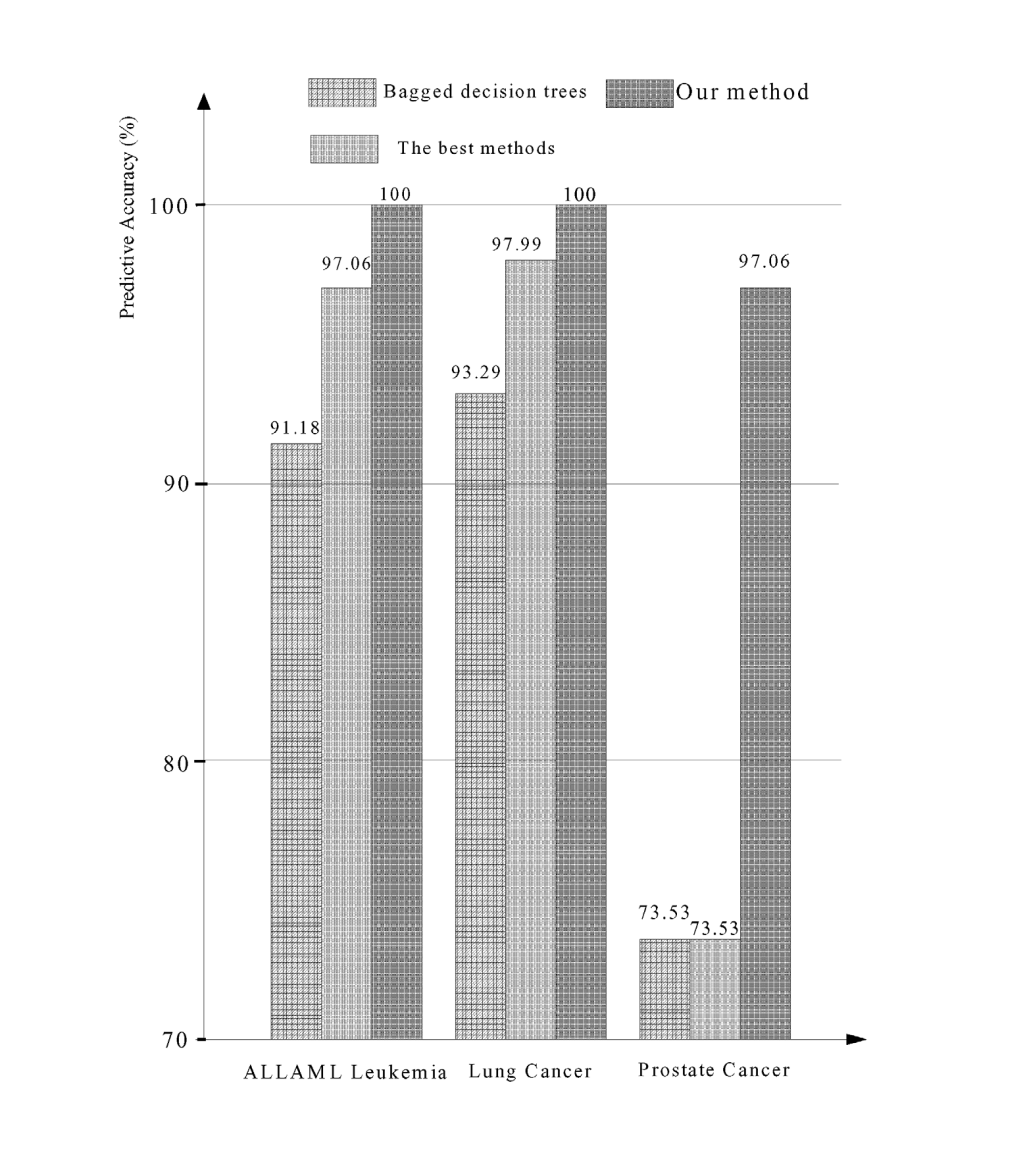
Comparing predictive accuracy of 3 separate testing samples with other methods

From the results of the above three testing datasets, we can also see that many different classifiers obtain the best results when they concern some certain dataset, but there is still no general best strategy for tumor classification problems based on a wide range of different datasets. Furthermore, from Figure 3, it is clear that our method is superior to the traditional bagging decision trees. Thus, we conclude that by using our method a more general accuracy improvement can be achieved for tumor classification.

#### The results of binary classification without testing samples

**Figure 4:**
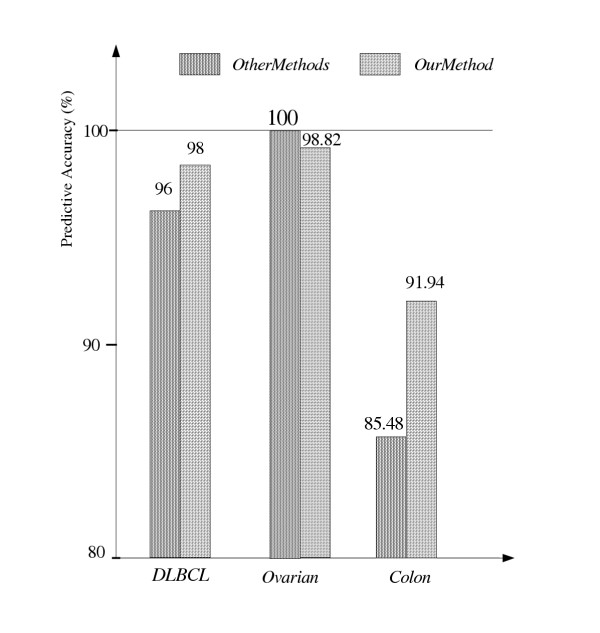
Comparing predictive accuracy of 3 datasets without testing samples with other methods

Without separate testing samples, we cannot evaluate the performance of our classifiers with the predictive accuracy of testing samples in the same way as above. Many performance evaluation methods have been proposed, of which various cross validations are most popularly used, such as 3-fold cross validation, 10-fold cross validation, leave-one-out cross-validation (LOOCV), and others. Here, we used the leave-one-out cross validation (LOOCV) to evaluate the performance of ours based on these available datasets. For further comparison with recent published methods based on the same datasets, we also perform 10-fold cross validation just as they used in their research. In Table 3, we list the predictive accuracy of our methods using 10-fold cross validation and LOOCV respectively and the corresponding results of other methods based on the same dataset and the same evaluation mechanism. These comparisons based on data in Table 3 are shown in Figure 4.

In the first data column of Table 3, we show our predictive accuracy 97.87% and 95.74% by LOOCV and 10-fold cross validation respectively. But unfortunately, we did not find the corresponding result based on this dataset. Cho et al. [25] artificially divide the dataset into 22 training samples and 25 test samples, and their best classification result is 96%. For the purpose of comparison, we also use the same strategy as Cho et al.'s [25] and in Figure 4 we can see that 98% predictive accuracy obtained by our method is a little better than theirs. O'Neill and Song [23] used neural network to get very good result based on the lymphoma dataset. But here the dataset we used is based on different subset and we can't compare our result with theirs.

For the ovarian dataset we used, Liu et al. [21] reported that 100% predictive accuracy can be obtained running 10-fold cross validation on all 253 samples under an all-χ^2 ^feature selection heuristic and support vector machine (SVM). In our method, only 75 features in total were used, and 99.21% and 98.82% accuracy was obtained respectively by LOOCV and 10-fold cross validation. Thus, we think that our method is comparable to theirs to some extent.

For the colon tumor dataset, we found that 85.48% predictive accuracy is the best classification result obtained in Dettling et al. [27], where they used various boosting algorithms and adopted leave-one-out cross validation (LOOCV). As shown on Figure 4, compared with our predictive accuracy by LOOCV, a significant accuracy improvement was obtained by using our method. Our result by using 10-fold cross validation also is shown in Table 3.

From the results of the above three datasets, we can see that our method is better, or at least comparable to current other best methods. Also, we need to note that these named best methods can get the best results based on certain datasets but may get worse results based on other datasets (Here we omitted the concrete comparisons on wide range datasets; correlated information can be found in our references); however, general performance improvement can be obtained using our method.

#### The result of multi-class problem

Finally, we generalize our method from binary class to multi-class problems. After minor adjustments to the corresponding parameters of our framework, we obtained 100% classification result of the above-mentioned multi-class dataset – MLL_Leukemia dataset, that is, 4 ALL, 3 MLL, 8 AML of 15 test data can be predicted correctly. Similarly, 100% accuracy also was obtained by Li et al. [28]. In this way, we conclude that our method also is fit to multi-class problems, and the classification result is comparable to other methods.

## Discussion

In this paper, we introduce a combinational feature selection and ensemble neural network method for the classification of gene expression data. On a wide range of recently published datasets, our method performs better, or is at least comparable to, the current best methods of our knowledge. As a further test, we randomly selected genes of the same amount as the feature instead of any of the three individual selected features in our research and then used the ensemble neural networks based on these features for classification again. The apparently worse discrimination power can be seen in this strategy. Moreover, we also used the output of a unitary network based on all the same features as the ultimate classification result and the result was also worse than ours. Thus, we believe such remarkable performance improvements of our method are due to the fact that our combinational feature selection mechanism induced more useful information for discrimination, and the ensemble neural network framework improved the stability, robustness and generalization of learning.

We performed simple majority voting mechanism to combine the individual networks produced by bagging and got a more accurate solution. The advantage of the ensemble is to reduce the variance, or instability of the neural network, and avoid the error surface of neural network training being trapped into local minima. The ensemble model tends to cancel the noise part as it varies among the ensemble members, and it tends to retain the fitting to the regularities of the data. In this paper, our ensemble neural network model has 100 members; However, further research is needed to determine how many members working together can reach the best performance.

In this paper, we focused on classification problem, so we didn't give a detailed analysis about how the importance of each different gene we select and the interaction between them influenced the diseases, which is a very important issue for application and will be researched in our future work.

Note that the only drawback of our approach is the problem of increasing computational complexity and the fact that it consumes a little more time than others. However, considering the lost caused by wrong prognosis or diagnosis of disease, we believe that the remarkable improvement in corresponding accuracy deserve these costs.

## Conclusions

By aggregating various information and ensemble neural networks, we reached a more accurate classification decision based on several datasets. We think that making full use of all available information will more clearly elucidate the latent mechanisms of many diseases. For example, we can combine various imaging techniques, such as CT, MRI, PET and others, which can detect the change of phenotype for the corresponding disease, with microarray data for further research. In this way, we can recognize the nature of various life phenomena both from macro and micro viewpoints. Also, we can retrieve the information of genes that are used in microarray, such as gene functions and gene locations. In this way, we can make use of prior knowledge combined with the microarray data for further research.

## Methods

### Feature selection

Feature selection is one of the most important issues in classification, which is a transformation process of observations in order to obtain the best pathway for getting to the optimal solution. At the same time, it can reduce the complexity of the data to make it more comprehensible. It is particularly relevant for microarray datasets with thousands of features because it has been reported that many diseases, especially tumors, have never been caused by a single gene mutation but are the result of a series of gene changes. Such genes are highly relevant to the studied phenomena of diseases. On the other hand, the expression levels of many other genes may be irrelevant to the distinction between tissue classes. We can say that the extraction and selection of features determine the ultimate performance of classifiers. Both for cost and for biological insight, making full use of the most informative genes and finding small feature sets with high classification accuracy are very essential. At the same time, highly informative genes that are part of known biochemical pathways give insights into the processes that underlie the differences between classes, and those of unknown function suggest new research directions.

Some classifiers, such as trees, perform automatic feature selection and are relatively insensitive to the variable selection scheme, but most classifiers need to perform feature selection first. So far, various feature selection schemes have been used in microarray data analysis, such as the most popular method of selecting the top-ranked genes based on various different scores (Euclidean distance, correlation coefficient, mutual information, signal to noise ratio) [9,11,22,25,29]. These feature selection methods gain better results on certain datasets, and the selective informative genes are the marker genes providing more useful information for further diagnosis and treatment. However, a problem with the above approaches is that they tend to select more correlated features so as not to provide more useful information for the purpose of classification. Li et al. [28] conclude that sometimes low-ranked genes are found to be necessary for classifiers to achieve perfect accuracy. It is conceivable that these useful low-ranked genes might have some relations with some important biological pathways and might have a vital influence on some diseases. Just selecting top-ranked genes will inevitably lose essential information. In order to compensate for this shortcoming, Jaeger et al. [22] proposed an improved gene selection for classification of microarrays. They demonstrated that the traditionally selected genes based on top-ranked scores are usually highly correlated, and they solved that problem through retrieving groups of similar genes first and then applying test-statistic to finally select genes of interest from these groups. In this way, the selected genes can correspond with some biological insights and might give out more accurate prediction about disease. The difficulty of this method lies in determining how many clusters and how many genes might directly correspond to the pathway on certain problems. Also, many researchers get the first several principle components by using PCA or SVD as the selected features, which captures most variation between samples and to some extent can obtain better results [12,30-33]. However, principle components cannot provide comprehensible rules to help elucidate the scheme of the related disease because it can be due to noise as well as true difference in expression and we do not know how many genes to pick.

Just as we alleged above, in such a high-dimension space, finding accurate and significant features (genes) is very essential for classification, for cost savings and for biological insights. However, it has been verified that differently selected features reflect different aspects of the dataset and some selected features can obtain better solutions on certain problems. This is because one feature selection mechanism corresponds to one different artificial hypothesis, but which hypothesis is most near to the true hypothesis on a special problem is unknown to us. Here, we propose combining the above mentioned several feature selection methods to reflect different profiles of samples in order to obtain more useful information for classification and to produce a good approximation to true hypothesis by averaging the different hypotheses. In this paper, we select features using wilcxon's ranksum test [34] to get the top-ranked genes, use PCA [31] to obtain the principle components as the feature, and use Jaeger's clustering method to group the whole genes into different clusters, and then select the top-ranked genes by t-test [34] scores from these groups. After picking these features from gene expression data, how to make full use of these features for further accurate classification is still a problem. Detailed illustration of our strategy is given below.

### Ensemble neural networks

An Artificial Neural Network (ANN) is an information-processing paradigm that is modeled on biological nervous system, which is composed of a large number of highly interconnected processing elements (neurons) working in unison to solve specific problems. In fact, since the basic model was proposed, various improved algorithms and theories have already been successfully applied in many fields. Because neural networks are best at identifying patterns or trends in a large amount of data with little theory, they are well suited for prediction or forecasting needs. That's just the case for microarray data. However, instability and little intrinsic knowledge of neural networks are obstacles to its further generalized application in some specific problems. Here we ensemble multiple networks in an attempt to solve this problem to some extent.

Since multi-net systems were introduced by Sharkey in 1996 [35], the combination of a number of neural networks has been widely applied in many fields. Because combining the outputs of several neural networks into an aggregate output often gives improved accuracy over any individual output, the objective of this kind of ensemble module is to solve problems that are difficult for a single neural network, and is to combine the individual outputs to achieve better generalization. Remarkable advantages of the ensemble compared with a unitary network have been demonstrated previously [36,37], one advantage of which is that it can to some extent ease the obstacles mentioned above and can improve the stability of neural network decisions.

Generally speaking, in neural networks ensemble, two problems need to be resolved: how to generate the individual network and how to combine them together. Using bootstrap or boosting resample mechanism to obtain the individual network is the most popular method to solve the first problem. Bootstrap is the most popular resample mechanisam of sampling with replacement, therefore some observations are duplicated and some are omitted. Boosting means to boost a "weak" learning algorithm into a "strong" learning algorithm. Their differences are that using bootstrap can resample uniformly and can get the individual network immediately, but boosting weights every sample in each iteration and must generate the individuals in sequence. Several recent published papers claimed that adaboost-the basic boosting algorithm is not fit to microarray data analysis [20,26], and some improved boosting have been made to increase the accuracy to some extent [26,27]. With no exception, they perform boosting in conjunction with decision trees. Here, we perform our resample mechanism using bagging, that is, bootstrap aggregating, which has been shown to work well in the presence of noise [24]. Due to the noisy fact of microarray data, here we use bagging to resample. As a further validation, we also used adaboost instead of bagging to resample in order to construct the individual network and the result is worse than bagging networks. As to the second problem, many ensemble mechanisms have been researched in recent years; for example, improvements in performance can result from training the individual networks to be decorrelated with each other [38] with respect to their errors. In this paper, we only adopt the majority voting, the basic ensemble method, to obtain the ultimate output result. Note that here we use the soft-voting mechanism, that is, the confidence of each net output is applied as voting value, rather than unit or zero.

Considering the complexity and noise of microarray data, it's very difficult to get a perfect solution using a unitary neural network based on some certain selected features. At the same time, accurately extracting and selecting the most informative genes is also very difficult using individual available methods. Thus, in this paper we attempt to combine multiple modes of information available from gene expression data using neural networks ensemble to get a better solution. We presented a cooperative and competitive neural network system that each of nets has the same architecture and topology, and each can respectively learn to classify a set of patterns based on partial information of the patterns and then by combining their classification results we can get a more precise result. The detailed framework of combing various features and neural networks ensemble and its implementation methods are discussed below.

## Authors' contributions

BL carried out the design of the method and performed the related analysis. QC participated in discussions of algorithms and manuscript preparation. TJ and SM instructed the whole study. All authors read and approved the final manuscript.

## Figures and Tables

**Table 1:** Gene expression datasets used in this paper

Dataset	Number of genes	Training samples	Testing samples	References
ALL-AML Leukemia	7129	38 (27:11)	34 (20:14)	Golub et al (1999)
Lung Cancer	12533	32 (16:16)	149 (15:134)	Gordon et al (2002)
Prostate Cancer	12600	102 (52:50)	34 (25:9)	Singh et al (2002)
DLBCL	4026	47 (24:23)	0	Alizadeh et al (2000)
Ovarian Cancer	15154	253 (91:162)	0	Petricoin et al (2002)
Colon Tumor	2000	62 (40:22)	0	Alon et al (1999)
MLL_Leukemia	12582	57 (20:17:20)	15 (4:3:8)	Armstrong et al (2002)

All these datasets are downloaded from

**Table 2:** The predictive accuracy of testing samples

	**ALLAML Leukemia**	**Lung cancer**	**Prostate cancer**
Bagged decision trees	**91.18%**	**93.29%**	**73.53%**
The best methods	**97.06%**	**97.99%**	**73.53%**
Our methods	**100%**	**100%**	**97.06%**
LOOCV on training samples	**100%**	**100%**	**96.08%**

* Note that the row of the best methods refer to the different method in different datasets

**Table 3:** The predictive accuracy by LOOCV and 10-fold CV

		DLBCL	Ovarian cancer	Colon tumor
LOOCV	Other methods	—	—	**85.48%**
	Our method	**97.87 %**	**99.21%**	**91.94%**
10-fold CV	Other methods	—	**100%**	—
	Our method	**95.74%**	**98.82%**	**90.32%**

* Note that the two rows of other methods refer to the different best method in different datasets
